# Periprosthetic infection is the major indication for TKA revision – experiences from a university referral arthroplasty center

**DOI:** 10.1186/s12891-018-2314-1

**Published:** 2018-11-10

**Authors:** S. P. Boelch, A. Jakuscheit, S. Doerries, L. Fraissler, M. Hoberg, J. Arnholdt, M. Rudert

**Affiliations:** Department of Orthopaedic Surgery, Julius-Maximilians University Wuerzburg, Koenig-Ludwig-Haus, 11 Brettreichstrasse, 97074 Wuerzburg, Germany

**Keywords:** Knee arthroplasty, Revision, Periprosthetic infection, Failure

## Abstract

**Background:**

We hypothesized, that periprosthetic joint infection (PJI) accounts for the major proportion of first (primary) and repeated (secondary) Total Knee Arthroplasty revisions at our university referral arthroplasty center.

**Methods:**

One thousand one hundred forty-three revisions, performed between 2008 and 2016 were grouped into primary (55%) and secondary (45%) revisions. The rate of revision indications was calculated and indications were categorized by time after index operation. The odds ratios of the indications for primary versus secondary revision were calculated.

**Results:**

In the primary revision group PJI accounted for 22.3%, instability for 20.0%, aseptic loosening for 14.9% and retropatellar arthrosis for 14.2%. PJI (25.6%) was the most common indication up to 1 year after implantation, retropatellar arthrosis (26.8%) 1–3 years and aseptic loosening (25.6%) more than 3 years after implantation.

In the secondary revision group PJI accounted for 39.7%, aseptic loosening for 16.2% and instability for 13.2%. PJI was the most common indication at any time of revision with 43.8% up to one, 35.4% 1–3 years and 39.4% more the 3 years after index operation.

The odds ratios in repeated revision were 2.32 times higher (*p* = 0.000) for PJI. For instability and retropatellar arthrosis the odds ratios were 0.60 times (*p* = 0.006) and 0.22 times (*p* = 0.000) lower.

**Conclusions:**

PJI is the most common indication for secondary TKA revision and within one year after primary TKA. Aseptical failures such as instability, retropatellar arthrosis and aseptical loosening are the predominant reasons for revision more than one year after primary TKA.

## Background

Total knee arthroplasty (TKA) is the treatment of choice for symptomatic arthrosis. Patient satisfaction with TKA has improved from 81.2% between the years 1990 and 1999 to 85% between the years 2000 and 2012 [1], but still absolute revision numbers are increasing. Although revision rates after TKA remain constantly low, data from the Nationwide Inpatient Sample (NIS) showed an increase of TKA revisions of 39% from 48,260 in 2006 to 67,534 in 2010 in the US [2]. In the first annual report of the German joint registry an increase of 144% was demonstrated from 7238 in 2004 to 17,658 in 2014 [3]. Recent clinical studies focusing solely on primary revisions found aseptic reasons such as instability with 19 and 22%, and aseptic loosening with 31 and 22% the two most common indications [4, 5]. The analysis of the Swedish, Norwegian, Finnish, Danish, Australian and the New Zealand registry by Sadoghi et al. stated, that the two most common reasons for TKA revisions between 1979 and 2009 were aseptic and septic loosening with 29.8% and 14.8%, respectively [6]. In contrast to aseptical revisions, management of periprosthetic joint infections (PJI) necessitates an interdisciplinary setting and special care [7]. This peculiarity of PJI management leads to a pooling of the affected patients at specialized referral arthroplasty centers, as the study institution is.

That is why we hypothesized, that PJI accounts for the major proportion of primary and secondary revisions at our institution. Additionally, we hypothesized that, in contrast to primary revision, the frequency of PJI is not related to time of revision for secondary revision.

## Methods

### Study design

This observational study was performed at the Department of Orthopaedic Surgery, University of Wuerzburg in Germany. Approval was waived by the University’s ethics committee (approval number 20180613 01).

### Setting

In August 2016, our department’s electronic data was scanned for all TKA revisions, that were performed since the introduction of our electronic database in December 2008. Only procedures involving an arthrotomy were considered a revision. The failure mechanism described by the operating surgeon as decisive for revision strategy was defined as indication for revision. Indications were categorized into polyethylene (PE)-wear, aseptic loosening, PJI, instability, periprosthetic fracture, malalignment, extensor mechanism deficiency, arthrofibrosis, retropatellar arthrosis and other.

The authors acknowledge that diagnostic algorithms of the painful TKA are discussed controversial and are still under investigation [8–12]. Thus, the predominant indications for revision are described in brief: PE-wear was diagnosed by radiographs showing osteolysis or progressive joint space narrowing under load, by intraoperatively macroscopic visible wear and by histopathologic evaluation of intraoperative samples according to Krenn and Morawietz [13].

PJI was evaluated in accordance with the guidelines of the Infection Disease Society of America [14]. Two stage exchanges were regarded as one event.

Instability was assessed based on the patient’s history for example with swelling and giving way events. Additionally, coronal and sagittal instability was evaluated by clinical examination and on radiographs as described elsewhere [10]. In cases of concomitant loosening or PE-wear, these were the primary diagnosis.

Arthrofibrosis was diagnosed by painful restriction of range of motion that was refractory to intensified physiotherapy, without any other underlying reason.

For alignment evaluation, we routinely used the alignment parameters based on The Knee Society Total Knee Arthroplasty Roentgenographic Evaluation and Scoring System [15, 16]. CT-scans were added on the bases of clinical and radiologic work up.

### Patients

One thousand one hundred forty-three revisions were identified. Revisions were performed in 36.4% in male and in 63.6% in female patients with a mean age of 67.9 years (21–93). First revisions after the primary TKA (index operation) were assigned to the primary revision group (55.0%). In case of any previous revision, which was not the primary implantation, this was regarded the index operation for the secondary revision group (45.0%).

The mean duration from index operation to revision was 42.1 months (0–279). 55.5% of the primary revision cases and 38.9% of the secondary revision cases were transferred to our institution for further operation.

To support a unification of the time to failure categorization we subdivided the time from index operation to revision in accordance with the recently published study by Thiele et al. into 1 year, 1–3 years and more than 3 years [5].

### Statistics

Means were compared with the t-Test. Odds ratios for the indications were calculated depending on primary or secondary revision and tested for significant differences with the Pearson chi square test. Statistics were performed with SPSS 24 (SPSS Inc. Chicago, USA).

## Results

### Primary revision group

**Fig. 1:**
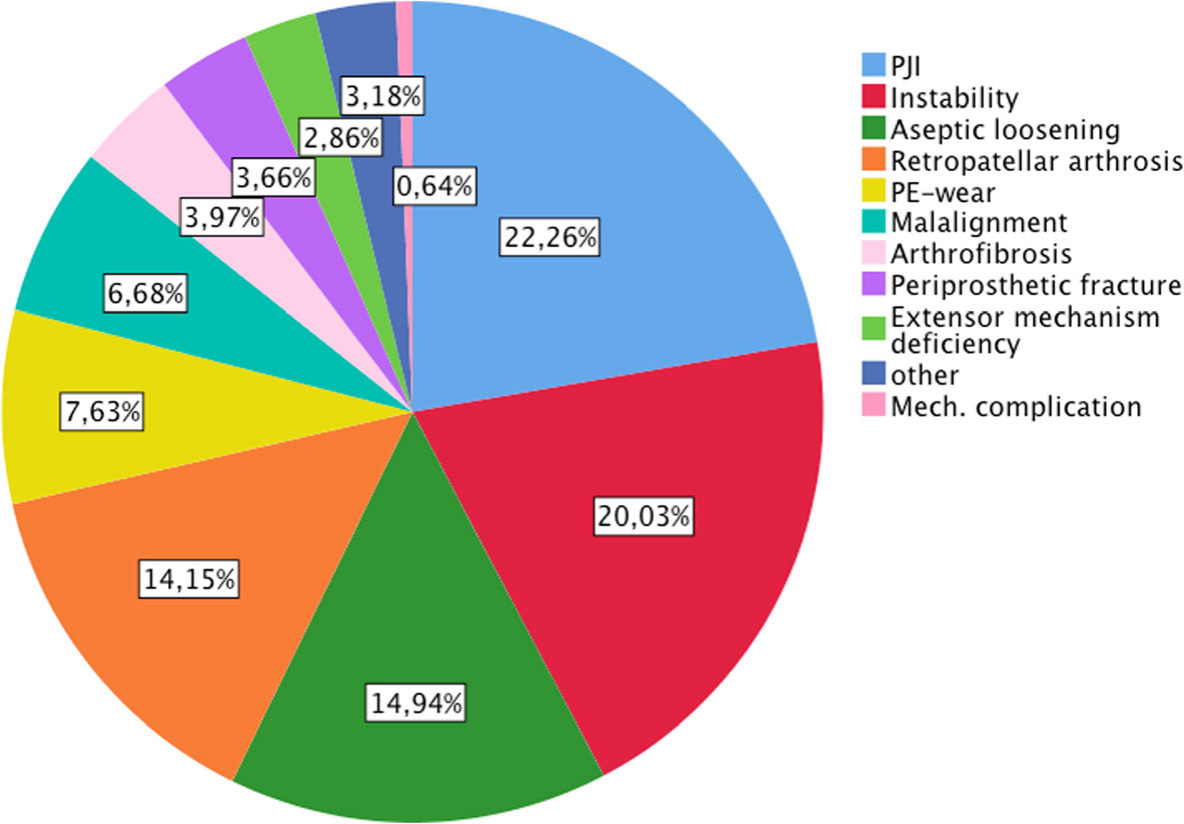
Indications for primary revision

In the primary revision group the major proportion (71.4%) of revisions was due to the four indications: PJI (22.3%), instability (20.0%), aseptic loosening (14.9%) and retropatellar arthrosis (14.2%) (Fig. 1).

**Fig. 2:**
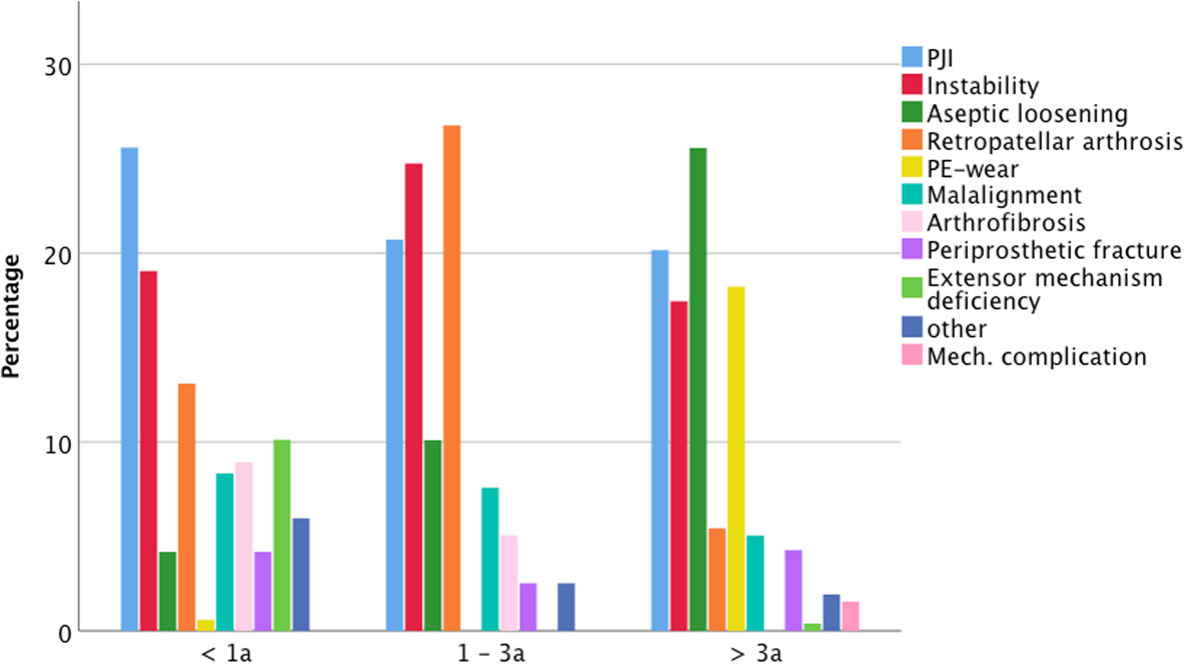
Distributions of indications for primary revision

26.7% were revised within 1 year after the implantation. In this group, the most common indication was PJI (25.6%), followed by instability (19.0%) and retropatellar arthrosis (13.1%). 31.5% of TKAs failed 1–3 years after implantation. Of these 26.8% were due to retropatellar arthrosis, 24.7% due to instability and 20.7% due to PJI. 41.0% were revised more than three after index operation. 25.6% of these revisions were because of aseptic loosening, 20.2% because of PJI, 18.2% because of PE-wear and 17.4% because of instability. The complete distributions are shown in Fig. 2.

### Secondary revision group

**Fig. 3:**
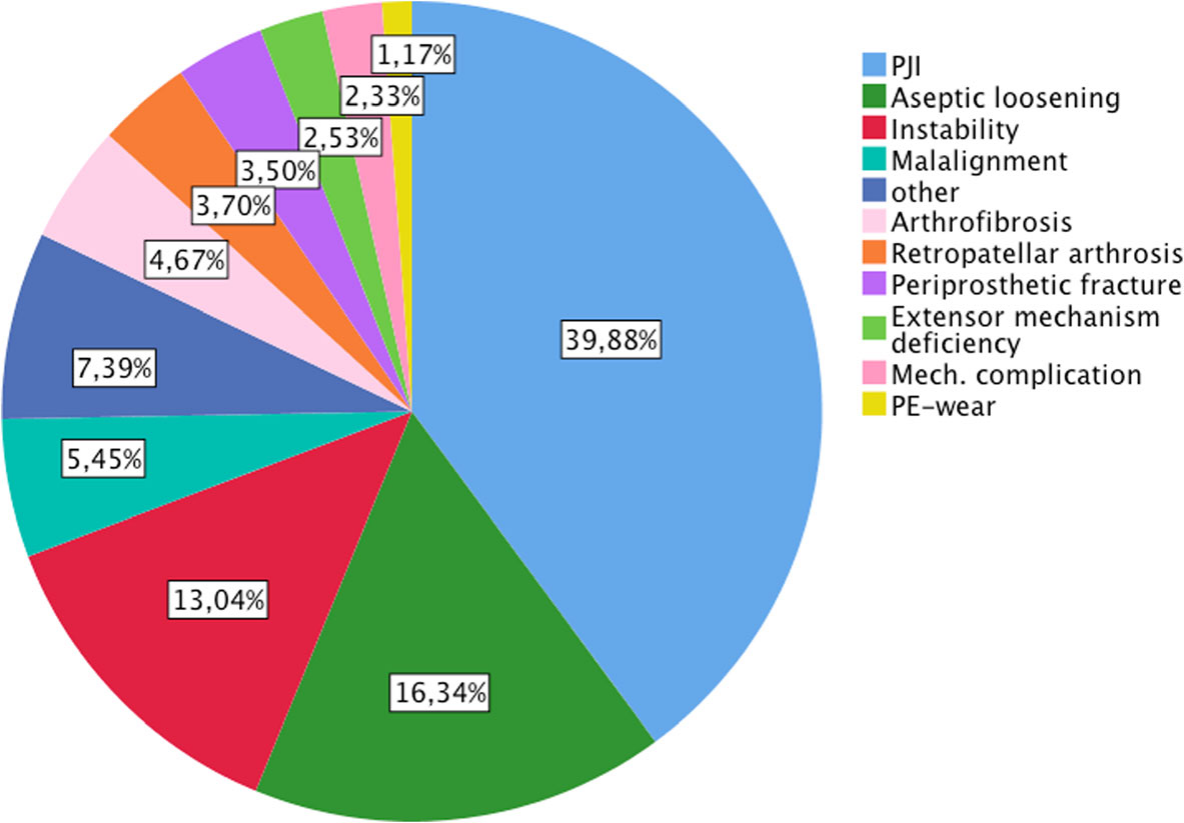
Indications for secondary revision

In the secondary revision group 68.1% of the revisions were due to three indications: PJI (39.7%), aseptic loosening (16.2%) and instability (13.2%) (Fig. 3).

**Fig. 4:**
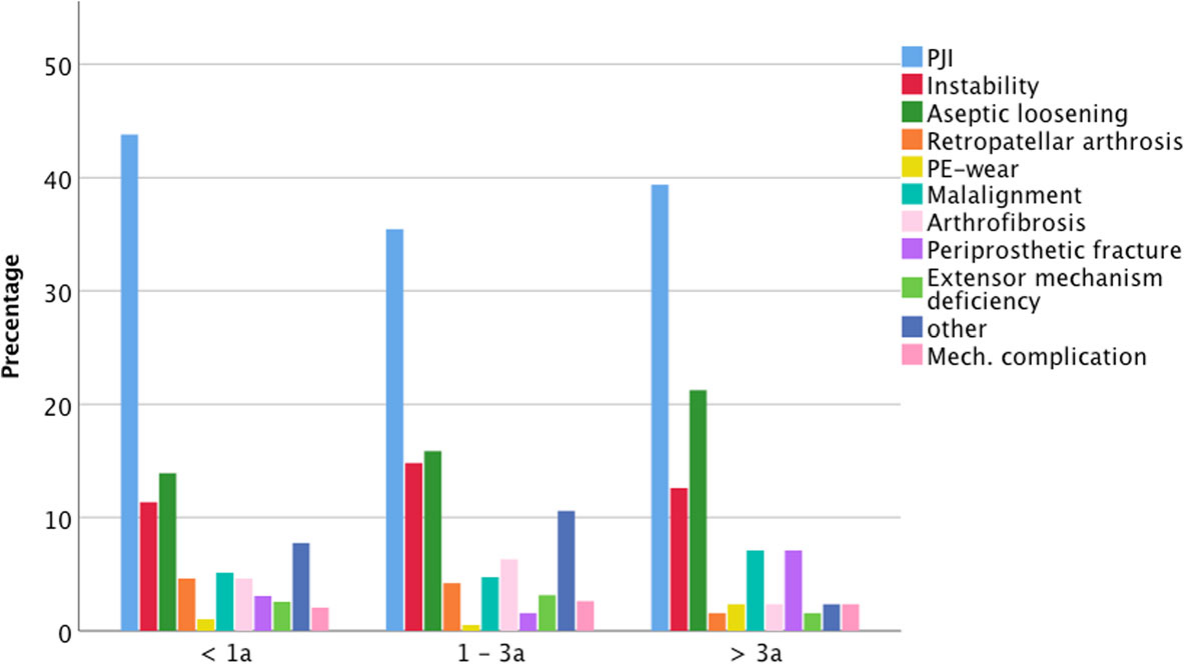
Distributions of indications for secondary revision

The most common indication of the 37.7% revisions within 1 year from index operation was PJI (43.8%), followed by aseptic loosening (13.9%) and instability (11.3%). A comparable distribution was found for the 36.8% revisions between 1 and 3 years with 35.4, 15.9 and 14.8% and for the remaining 25.5% revision after more than 3 years with 39.4, 21.3 and 12.6%. The detailed distributions are depicted in Fig. 4.

### Comparison of primary and secondary revisions

The odds of being revised for PJI were 2.5 times higher (*p* < 0.000) for secondary revisions. However, the odds of being revised for instability or retropatellar arthrosis were significantly lower for secondary revisions (Table 1).

**Table 1:** Odds ratios depending on secondary revision/primary revision of the 4 major indications for revision

Indication for revision	Odds ratio (95% CI)	Pearson chi square test p
PJI	2.32 (1.79–3.00)	0.000
Instability	0.60 (0.43–0.83)	0.002
Aseptic loosening	1.11 (0.81–1.53)	0.517
Retropat. arthrosis	0.22 (0.13–0.36)	0.000

The mean duration to revision because of PJI and because of aseptic loosening was significantly (*p* = 0.000) shorter in the secondary revision group (Table 2).

**Table 2:** Mean duration and range from index operation to revision in months

Indication for revision	Duration to primary revision in months (range)	Duration to secondary revision in months (range)	*p*
PJI	49.00 (0–279)	28.11 (0–198)	0.000
Instability	37.67 (2–226)	31.48 (4–153)	0.301
Aseptic loosening	94.68 (5–242)	31.05 (1–163)	0.000
Retropatellar arthrosis	24.54 (3–106)	19.53 (3–58)	0.677

## Discussion

We found PJI to be the most common indication for both, primary and secondary TKA revision at a university referral arthroplasty center. This result is in accordance with the numbers published from the NIS for knee arthroplasty revisions, without discriminating primary from secondary revisions [17]. The odds ratio from the current study demonstrates, that PJI is particularly the major revision indication for secondary revisions. The few other available studies on reasons for re-revisions report comparable rates of PJI as revision indication for secondary revisions. Suarez et al. had a re-revision rate of 46% for PJI in their 68 knees that underwent secondary revision [18]. Mortazavi et al. described this rate to be 44% in their study of 102 knees [19]. However, in contrast to previous publications, we found PJI to be the most common indication for primary revisions, too. In the study of 358 primary revisions by Thiele et al. PJI was the fourth most common indication with a proportion of 15% [5]. In their study, revisions with component retention were excluded. However, debridement and irrigation with retention of the fixed components is a warranted treatment regime for early postoperative or acute periprosthetic infection [14, 20]. These cases are included in our study and are represented by the finding, that PJI was predominantly found for primary revision within 1 year after index operation. Schroer et al. described PJI the third most common reason with a proportion of 16.2% of 844 patients treated at six different institutions [4]. However, we present monocentric results based on standardized diagnostic algorithms.

In contrast to PJI, retropatellar arthrosis and instability are specific issues of primary TKA. In accordance with our results, instability is consistently reported a major failure mechanism after primary TKA [4, 5, 21]. However, retropatellar arthrosis was the most common revision reason 1–3 years after the implantation, what reflects the development of clinically relevant and radiographically obvious retropatellar wear.

This study has limitations because of its retrospective design and the complexity of TKA revision.

If the treatment of PJI failed and the patient was readmitted, the following revision was considered a new case. In this study with 345 primary and secondary revisions, about 10% were repeatedly revised for reinfection. Thus, the proportion of PJI might be biased by patient specific characteristics.

Further, we did not investigate the proportion of resurfaced patellae before revision. These cases were not excluded from the analysis because the proportion of failures due to patellar arthrosis is clinically relevant and the discussion of the best treatment is still going on [22]. The significantly higher proportion of retropatellar arthrosis in the primary revision group is highly likely to be owed to the fact, that the secondary revisions had a higher rate of resurfaced patella before re-revision.

The 14th NJR annual reported aseptical failure mechanisms the most frequent reasons for primary revision [23]. The current monocenter study at a university referral arthroplasty center found PJI the leading failure mechanism irrespectively whether after primary or revised TKA. This discrepancy is owed the pooling of patients. It displays the enormous challenge of referral arthroplasty centers to especially ensure the management of PJI as a potentially life threating TKA failure with the danger of devastating sequela.

## Conclusion

PJI is the most common indication for secondary TKA revision and within one year after primary TKA. Aseptical failures such as instability, retropatellar arthrosis and aseptical loosening are the predominant reasons for revision more than one year after primary TKA.

## Abbreviations

CT: Computed tomography
NIS: Nationwide Inpatient Sample
PE: Polyethylene
PJI: Periprosthetic joint infection
TKA: Total knee arthroplasty
